# Chromosome-scale genomics, metabolomics, and transcriptomics provide insight into the synthesis and regulation of phenols in *Vitis adenoclada* grapes

**DOI:** 10.3389/fpls.2023.1124046

**Published:** 2023-01-25

**Authors:** Guo Cheng, Daidong Wu, Rongrong Guo, Hongyan Li, Rongfu Wei, Jin Zhang, Zhiyong Wei, Xian Meng, Huan Yu, Linjun Xie, Ling Lin, Ning Yao, Sihong Zhou

**Affiliations:** ^1^ Grape and Wine Research Institute, Guangxi Academy of Agricultural Sciences, Nanning, China; ^2^ Bureau of Agriculture and Rural Affairs of Luocheng Mulao Autonomous County, Hechi, China; ^3^ Guangxi Luocheng Maoputao Experimental Station, Hechi, China

**Keywords:** *Vitis adenoclada*, phenols, genomics, metabolomics, transcriptomics, metabolic regulation

## Abstract

*Vitis adenoclada* is a wild grape unique to China. It exhibits well resistance to heat, humidity, fungal disease, drought, and soil infertility. Here, we report the high-quality, chromosome-level genome assembly of GH6 (*V. adenoclada*). The 498.27 Mb genome contained 221.78 Mb of transposable elements, 28,660 protein-coding genes, and 481.44 Mb of sequences associated with 19 chromosomes. GH6 shares a common ancestor with PN40024 (*Vitis vinifera*) from approximately 4.26–9.01 million years ago, whose divergence occurred later than *Vitis rotundifolia* and *Vitis riparia*. Widely-targeted metabolome and transcriptome analysis revealed that the profiles and metabolism of phenolic compounds in *V. adenoclada* varieties significantly were differed from other grape varieties. Specifically, *V. adenoclada* varieties were rich in phenolic acids and flavonols, whereas the flavan-3-ol and anthocyanin content was lower compared with other varieties that have *V. vinifera* consanguinity in this study. In addition, ferulic acid and stilbenes content were associated with higher expressions of COMT and STSs in *V. adenoclada* varieties. Furthermore, MYB2, MYB73-1, and MYB73-2 were presumably responsible for the high expression level of COMT in *V. adenoclada* berries. MYB12 (MYBF1) was positively correlated with PAL, CHS, FLS and UFGT.Meanwhile, MYB4 and MYBC2-L1 may inhibit the synthesis of flavan-3-ols and anthocyanins in two *V. adenoclada* varieties (YN2 and GH6). The publication of the *V. adenoclada* grape genome provides a molecular foundation for further revealing its flavor and quality characteristics, is also important for identifying favorable genes of the East Asian species for future breeding.

## 1 Introduction

*Vitis adenoclada* is a wild grape species native to China that is commonly distributed in Hunan, Fujian, Guangxi, and other provinces south of the Yangtze River. It belongs to the East Asian population of *Vitis* spp.–Maoputao group (Niu and He, 1996). Of note, *V. adenoclada* is easily confused with *Vitis heyneana*. Actually, *V. adenoclada* possesses unique purplish brown glandular hairs on the new shoots and old mature vines (Wang, 1988) while *V. heyneana* does not, which is the biggest biological difference between them (Kong, 2004; Liu C. H., 2012). There was also study indicated that *V. adenoclada* should be downgraded into a variety of *V. heyneana* (Xie et al., 2021). According to field observations, the presence and density of glandular hairs varies with variety, habitat, nutrition, and other factors in *V. adenoclada* (**Supplementary Figure 1**). Guangxi Province is located in Southern of China, and it is one of the original locations of the East Asian population of *Vitis* spp. The results of the Third National Crop Germplasm Resources Survey in China revealed that *V. heyneana*, *V. adenoclada*, *Vitis davidii*, and *Vitis pseudoreticulata* are widely distributed in the region (Chen et al., 2020). There is a long history of viniculture by using wild grape species in Guangxi, and *V. adenoclada* grape is an important raw material for wine making in local area (Cheng et al., 2018). Since 2011, research has been carried out to breed the *V. adenoclada* varieties. Thus far, a series of excellent *V. adenoclada* varieties have been established and designated the “Guiheizhenzhu” series (Wu et al., 2020). They adapt to the climate and environmental conditions of the south tropical and subtropical regions, and exhibit resistance to heat and humidity, fungal diseases and pests, drought, and soil infertility (Wu et al., 2020).

Phenolics are important compounds that affect the sensory quality of grapes, and subsequently wine. The composition and content of phenolic compounds are determined by genotype (species/variety), ecological conditions, viticulture practice and other factors (Sun et al., 2019). In addition to the reported structural genes, the synthesis of these compounds is also regulated by many transcription factors, such as MYB, bHLH, WRKY, AP2/EREBP, C2C2, NAC, and C2H2 (Sun et al., 2017). In recent years, more and more studies have been conducted on the regulatory mechanism of phenolic metabolism in different grape varieties using multi-omics methods (Sun et al., 2019; Ju et al., 2020; Yang et al., 2020). Since 2007, a highly homozygous genotype, the inbred Pinot noir line, PN40024 (*Vitis vinifera*), has been sequenced, marking the beginning of the genomics era in grape research (Jaillon et al., 2007). Subsequently, genomes sequencing of different varieties have been carried out to better serve for research in *Vitis* genus, such as *V. vinifera* of Sultanina (Di Genova et al., 2014), Cabernet Sauvignon (Chin et al., 2016) and Chardonnay (Zhou et al., 2019), *Vitis riparia* (Patel et al., 2020), *Vitis arizonica* (Massonnet et al., 2020), *Vitis rotundifolia* (Cochetel et al., 2021; Park et al., 2022), *Vitis amurensis* (Wang et al., 2021). However, there are few reports about wild grape resources, especially focused on the quality characteristics and metabolic mechanism of related varieties using these self-testing genomes combined with transcriptome and metabolome.

In this study, we established a high-quality *de novo* genome assembly of GH6 (*V. adenoclada*). We created a chromosome-level assembly with an overall scaffold length of 498.27 Mb that included 28,660 annotated genes using a combination of Illumina and Oxford Nanopore Technologies (ONT) sequencing data and high-throughput chromosome conformation Capture (Hi-C) mapping. Notably, we also used this self-testing genome to examine the metabolism of phenolics among the different grape varieties. Through a combination of phenolic-associated metabolic studies and transcriptome analysis, we constructed a regulatory network of biosynthesis of resveratrol, phenolic acid, flavonol, flavan-3-ol, and anthocyanin. Furthermore, we identified key transcription factors that modulate phenolic metabolism using transcription factor prediction and co-expression network analyses. Overall, the established genome sequence is not only important for understanding the quality characteristics of the *V. adenoclada*, but it will also contribute to the further development and utilization of East Asian grape resources.

## 2 Materials and methods

### 2.1 Plant material, berry sampling, and physical chemical index analysis

The experimental location was in the vineyards of the Guangxi Academy of Agricultural Sciences’ Grape and Wine Research Institute in Nanning, Guangxi Province. The materials included eight varieties (**Supplementary Figure 2**): Cabernet Sauvignon (*V. vinifera*, CS), Marselan (*V. vinifera*, Mar), Petit Verdot (*V. vinifera*, PV), NW196 (*V. heyneana* × *V. vinifera*), Yeniang No.2 (*V. adenoclada*, YN2), Guiheizhenzhu No.4 (*V. adenoclada*, GH4), Guiheizhenzhu No.5 (*V. adenoclada*, GH5), and Guiheizhenzhu No.6 (*V. adenoclada*, GH6). Furthermore, YN2, GH4 and GH6 are bisexual flower varieties, whereas GH5 is a unisexual flower variety.

For genome sequencing and assembly, the GH6 plant was employed. The young fresh GH6 leaves were collected and swiftly frozen in liquid nitrogen. In addition, other young fresh GH6 leaves were collected, sliced using sharp blades, and fixed in a 2% formaldehyde solution at room temperature for 90 minutes before the cross-linking reaction being stopped by the addition of 2.5 M glycine. The tissues were treated for Hi-C library creation after being frozen in liquid nitrogen.

At harvest, berries from eight varieties in three biological replicates were gathered. 120 berries were randomly selected from at least 30 clusters within 9 vines for each biological replicate. After being transported to the laboratory, a subsample of 50 berries from each biological replicate were measured for fresh weight, pH, total soluble solids (TSS), and titratable acidity (TA) content. The leftover berries were flash-frozen in liquid nitrogen and stored at -80°C for further metabolomic and transcriptomic analyses.

### 2.2 Genome survey and sequencing

High-quality genomic DNA from GH6 was extracted *via* a CTAB-based protocol. DNA libraries with fragment lengths of about 350 bp were created using the Illumina-provided standard protocols. The libraries were sequenced in paired-end mode on an Illumina Novaseq 6000 platform with read lengths of 150 bp. The sequencing results were used to assess the genomic parameters of GH6 *via* K-mer analysis, such as genome size, GC content, heterozygosity, and the frequency of repeat sequences.

ONT’s standard protocol was followed for genome sequencing. To summarize, genomic DNA was randomly disrupted, and large DNA fragments were collected *via* the BluePippin device. The SQK-LSK109 kit was used to generate DNA libraries. Fragmentation, end repair, ligation of sequencing adapters, and magnetic bead purification were all performed on DNA fragments. Following that, DNA sequencing was carried out on the PromethION platform. All genome sequencing procedures were conducted by the Biomarker Technologies Corporation (Beijing, China).

### 2.3 Genome assembly and assessment

Raw Nanopore data were formatted, sequencing adapters were removed, and low-quality or short-length (<2000 bp) reads were filtered. After corrected using Canu (Koren et al., 2017), WTDBG (https://github.com/ruanjue/wtdbg) was used to assemble nanopore readings into contigs. The assembled contigs were further calibrated using Racon (Vaser et al., 2017) with two iterations and then polished using four iterations of Pilon (Walker et al., 2014) with the Illumina sequencing reads. Assembly quality was assessed based on three ways: CEGMA (Parra et al., 2007) (v2.5) and BUSCO (Simão et al., 2015) (v2.0) were used to examine the fullness of the core genes; Illumina sequencing data were mapped to the assembled genome using BWA (Li and Durbin, 2009) to estimate the mapping rates.

### 2.4 Construction of a Hi-C library and chromosomal assembly

Hi-C fragment libraries with a 300-700 bp insert size were constructed following the protocols described by Rao et al. (2014), then sequenced with the Illumina platform. To summarize, raw read adapter sequences were trimmed, and low-quality PE reads were deleted to clean the data. The clean Hi-C reads were first trimmed at the putative Hi-C junctions, and the trimmed reads were then BWA (Li and Durbin, 2009) (v0.7.10-r789) aligned to the assembly results. Invalid read pairs containing dangling-ends, self-cycles, re-ligation, and dumped products were removed by HiC-Pro (Servant et al., 2015) (v2.8.1). Only uniquely mapped read pairs were retained for assembly using LACHESIS (Burton et al., 2013). Following this procedure, the placement and orientation abnormalities that indicated clear discrete chromatin interaction patterns were manually corrected.

### 2.5 Genome annotation

Firstly, ab initio prediction for the repeat sequences was performed by using RepeatModeler2 (Flynn et al., 2020) (v2.0.1) with the softwares of RECON (Bao and Eddy, 2002) (v1.0.8) and RepeatScout (Price et al., 2005) (v1.0.6), then RepeatClassifier (Flynn et al., 2020) with database of Dfam (Wheeler et al., 2013) (v3.5) was used to classify the results of the prediction. Secondly, long terminal repeats (LTRs) were predicted based on the ab initio principle by using LTR_retriever (Ou and Jiang, 2018) (2.9.0) with LTRharvest (Ellinghaus et al., 2008) (v1.5.10) and LTR FINDER (Xu and Wang, 2007) (v1.07). It was then merged with all above predicted outcomes as the final repeat sequence database. RepeatMasker (Tarailo-Graovac and Chen, 2009) (v4.1.2) was used to predict the transposable elements (TEs) of GH6 based on the constructed repeat sequence database.

The prediction of protein-coding genes of the GH6 genome was done *via* three different strategies namely: ab initio prediction, homologous prediction, and RNA-seq prediction. Ab initio prediction was performed using Genscan (Burge and Karlin, 1997), Augustus (Stanke and Waack, 2003) (v2.4), GlimmerHMM (Majoros et al., 2004) (v3.0.4), GeneID (Blanco et al., 2007) (v1.4), and SNAP (Korf, 2004) (v2006-07-28). The homologous prediction of protein-coding genes based on other species (*V. vinifera*, *Z. jujuba*, *A. thaliana*, *O. sativa*) was done using GeMoMa (Keilwagen et al., 2016; Keilwagen et al., 2018) (v1.3.1). HISAT (Kim et al., 2015) (v2.0.4) and Stringtie (Pertea et al., 2015) (v1.2.3) were employed for assembly based on RNA-seq data with reference transcripts, then gene prediction was performed with TransDecoder (v2.0) (http://transdecoder.github.io) and Genemarks-T (Tang et al., 2015) (v5.1). Meanwhile, the prediction of Unigene sequences through the unreferenced assembly of RNA-seq data was performed with PASA (Campbell et al., 2006) (v2.0.2). Lastly, the prediction results of the above three methods were amalgamated *via* EVM (Haas et al., 2008) (v1.1.1). The predicted gene sequences were labelled annotations with functional databases including NR (Marchler-Bauer et al., 2011), KOG (Tatusov et al., 2001), KEGG (Kanehisa and Goto, 2000), and TrEMBL (Boeckmann et al., 2003) by BLAST (Altschul et al., 1990) (v2.2.31). Functional annotation of GO (Dimmer et al., 2012) was performed with Blast2GO (Conesa et al., 2005).

### 2.6 Comparative genomic analyses

Using Orthofinder (Emms and Kelly, 2019) (v2.4), protein sequences from *V. adenoclada* and nine other representative species were obtained for gene family clustering. The resulting gene families were further annotated using the PANTHER (Mi et al., 2019) (v15) database. Using the maximum likelihood approach and IQ-TREE (Nguyen et al., 2015) (v1.6.11), single-copy protein sequences were utilized to build a phylogenetic tree for *V. adenoclada* and the other nine species. The root was set to *A. trichopoda*, and the number of bootstraps was set to 1000. Subsequently, the divergence times were estimated using MCMCTREE (Puttick, 2019) in the PAML (Yang, 1997) package (v4.9i) and calibrated using the TimeTree (Kumar et al., 2017) website (http://www.timetree.org/). Based on the phylogenetic tree with divergence times and gene family clustering, the gene family expansion and contraction analysis were performed by CAFE (Han et al., 2013) (v4.2). The gene family members from the ancestor of each branch were estimated using the birth mortality model, which was applied to infer the contraction and expansion of the gene families (*p*-values<0.05). PANTHER was used to annotate the expanded and contracted gene families identified in *V. adenoclada*, and ClusterProfile was used to perform GO enrichment analyses on these families (Yu et al., 2012).

MUMmer (Marcais et al., 2018) (v4.0.0rc1) was used to identify the collinear blocks of two species genomes. Subsequently, visualization of Genome collinearities between *V. adenoclada* and the other three grape species of *V. vinifera*, *V. riparia*, and *V. rotundifolia* was performed by NGenomeSyn (https://github.com/hewm2008/NGenomeSyn). Using Diamond (Buchfink et al., 2015) (v0.9.29.130), the gene sequences of two species were compared and comparable gene pairs were determined. *V. adenoclada* was compared with *V. vinifera*, *V. riparia*, and *V. rotundifolia*. Genomes of *V. adenoclada*, *V. vinifera*, *V. riparia*, *V. rotundifolia*, and *Z. jujuba* were used for WGD analyses. Based on the distribution of 4DTv rate, which was estimated using the HKY model (Hasegawa et al., 1985) and a Perl script (https://github.com/JinfengChen/Scripts), WGD events were determined.

### 2.7 Widely-targeted metabolomic analysis

Metabolites detection was performed with the help of Metware Biotechnology Co., Ltd. (Wuhan, China). A vacuum freeze-dryer was used to freeze-dry eight fruit samples with three biological replicates. Lyophilized powder (100 mg) was dissolved in 1.2 ml of 70% methanol solution, vortexed for 30 seconds every 30 minutes for a total of 6 times, and stored in a refrigerator overnight at 4°C. After centrifugation at 12,000 rpm for 10 minutes, the extracts were filtered (SCAA-104, 0.22 µm pore size; ANPEL, Shanghai, China), then analyzed by UPLC-ESI-MS/MS. The analytical conditions, raw data preprocessing, basis data analysis, KEGG annotation, and metabolic pathway analyses of differential metabolites all referred to the previous report (Wang et al., 2020). The mass spectrometry data was processed using Analyst 1.6.3 software (AB Sciex, Framingham, MA, USA). The identified metabolites were annotated based on the KEGG compound database (http://www.kegg.jp/kegg/compound/) and then mapped to the KEGG pathway database (http://www.kegg.jp/kegg/pathway.html).

### 2.8 RNA extraction, library construction, and sequencing

RNA extraction, library creation, and sequencing were done according to a previously reported method (Fu et al., 2021), and Illumina sequencing was carried out by the Gene Denovo Biotechnology Co. Ltd. (Guangzhou, China). To guarantee high-quality clean reads for further assembly and analysis, reads were filtered by fastp (Chen et al., 2018) (v0.18.0). The parameters were chosen to eliminate adapter-carrying reads, reads having more than 10% unknown nucleotides (N), and low-quality reads containing more than 50% low-quality (Q-value ≤20) bases. The short read alignment tool, Bowtie 2 (Langmead and Salzberg, 2012) (v2.2.8), was used for mapping reads to the ribosomal RNA (rRNA) database. The mapped rRNA readings were then deleted and the remaining clean reads were used for assembly and calculating gene abundance. HISAT (Kim et al., 2015) (v2.2.4) was used to map the clean reads to self-assembled genomes. StringTie (Pertea et al., 2015; Pertea et al., 2016) (v1.3.1) was used to assemble the mapped reads for each sample using a reference-based technique. Using RSEM (Li and Dewey, 2011), an FPKM (fragment per kilobase of transcript per million mapped reads) value was computed for each transcriptional domain to evaluate its expression abundance and variation. All transcripts were annotated using databases such as GO, KEGG, NR, and Swiss-Prot, and RNA differential expression was analyzed using DESeq2 (Love et al., 2014) between two groups. DEGs or transcripts were defined as genes or transcripts with an FDR less than 0.05 and an absolute fold-change ≥2.

### 2.9 Transcription factor (TF) analysis

Considering the important role of TFs in the synthesis of phenols, the TFs expressed in all samples were identified. All putative TFs were retrieved by the predicted protein sequences compared with the plant TF database (TFdb) using hmmscan. For structural genes of the same family, the highest expression amount of gene with similar expression pattern was analyzed by clustering screening. Furthermore, a co-expression analysis was done between the phenolic biosynthetic pathway genes and TF genes (correlation coefficient >0.85). Networks were visualized using Cytoscape (Shannon et al., 2003) v.3.7.1.

### 2.10 Weighted gene co-expression network analysis

The overlapping DEGs and DAPs (differentially accumulated phenolics) were selected for co-expression network analysis using the WGCNA (v1.47) package in R (Langfelder and Horvath, 2008). More than half of the samples with genes of low abundance (FPKM value < 0.8) were filtered out to decrease the interference in the network analysis. The co-expression modules were obtained using the automatic network construction function (blockwiseModules) with default parameters, apart from the soft threshold power of 10, TOM type was signed, merge CutHeight was 0.6, and the minModuleSize was 50. After the initial module division, we obtained the Dynamic Tree Cut of the preliminarily divided module. Because some modules are very similar, we also merged the modules with similar expression modes according to the similarity of module eigenvalues to obtain 9 merged modules. Furthermore, the correlation coefficients between the hub genes in the module and the DAPs were calculated using OmicShare tools.

## 3 Results

### 3.1 Sequencing, assembly, and quality assessment of genome

**Figure 1:**
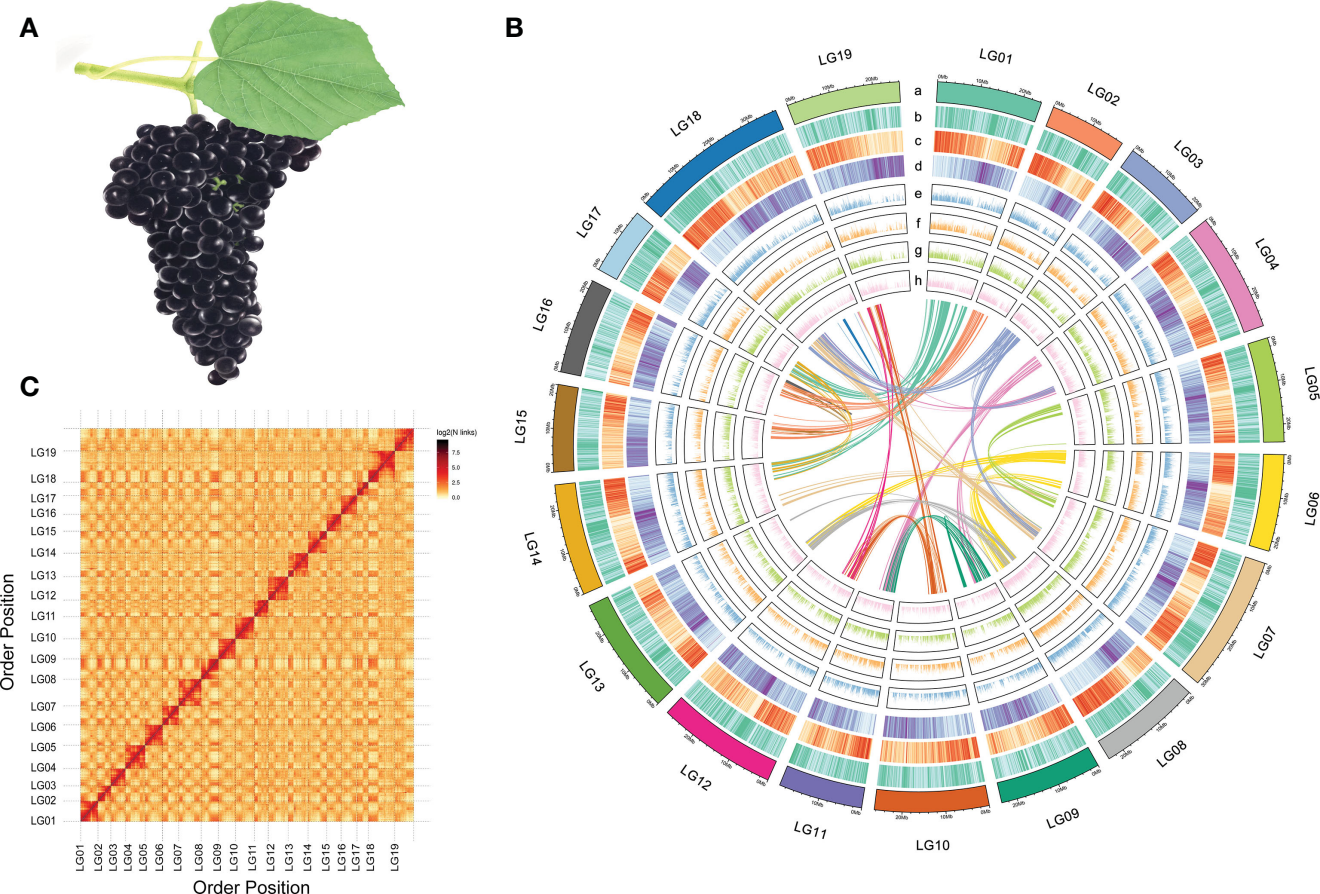
Morphology and genome information of *V. adenoclada*. **(A)** Drawing of *V. adenoclada* specimens. **(B)** Landscape of the *V. adenoclada* genome. a: chromosome ideograms; b: GC density; c: gene density; d: TE density; e~h: gene expression levels of YN2, GH4, GH5, and GH6, respectively; center: syntenic blocks within the genome. **(C)** Hi-C interaction heatmap of *V. adenoclada* genome.

The GH6 plant, a variety of *V. adenoclada* (**Figure 1A**), was chosen for genome sequencing and assembly. A genome survey was performed to analyze the Illumina sequencing data. According to a K-mer analysis, the approximate genome size of GH6 was 524.23 Mb, with a heterozygosity of 0.62% and a repeat sequence proportion of 49.18% (**Supplementary Table 1**). The ONT platform generated a total of 103.24 Gb of raw data. After cleaning, 94.99 Gb clean reads were obtained with a mean read length of 25.87 kb. Nanopore sequencing clean reads were subjected to genome assembly, calibrating, and polishing. The draft genome assembly size for GH6 was 498.21 Mb with a contig N50 of 2.91 Mb (**Supplementary Table 2**).

A total of 54.56 Gb data sequenced using the Illumina platform were used to construct a chromosome-level genome assembly for GH6. After assessing and filtering the paired-end reads, valid interaction pairs were applied to facilitate the Hi-C assembly (**Supplementary Table 3**). As a result, 481.44 Mb sequences could be anchored to chromosomes, accounting for 96.63% of the contig genome assembly (**Supplementary Table 4**). The resulting contigs were clustered into 19 pseudochromosomes (**Figure 1B**), of which 462.46 Mb (96.06%) could be verified by order and direction (**Supplementary Table 5**). Ultimately, the final chromosome-scale genome assembly of GH6 was 498.27 Mb with a scaffold N50 of 25.26 Mb (**Supplementary Table 4**). A chromosomal interaction heatmap was created to demonstrate a pattern consistent with the Hi-C genome assembly and to confirm the pseudochromosome construction (**Figure 1C**).

The assembly quality and completeness of the GH6 genome was assessed by two methods, CEGMA and BUSCO. CEGMA analysis indicated that the assembled genome fully recalled 422 (92.14%) of the 458 core eukaryotic genes (CEGs) and 184 (74.19%) of the 248 extremely conserved CEGs. BUSCO analysis revealed that 1463 (90.64%) of the 1614 orthologs from the Embryophyta dataset were fully captured in the assembly (**Supplementary Table 6**). In addition, when Illumina sequencing data were mapped to the assembled genome, the mapping rate was 96.97% (the proper mapping rate was 92.15%) against the genome assembly.

### 3.2 Genome annotation

TE is one main type of the repetitive sequences. The fully assembled genome of GH6 contained 221.78 Mb (44.51%) of TEs which distributed in 19 chromosomes (**Figure 1B**). LTR retrotransposons, which included Gypsy repeats (10.05%) and Copia repeats (10.41%), were the most prominent class of repetitive sequences (**Supplementary Table 7**). There were 28,660 protein-coding genes predicted with a full length of 150.75 Mb that were randomly distributed across the 19 chromosomes. A total of 27,711 (96.69%) genes were labelled functional annotations by BLAST using the GO, KEGG, KOG, TrEMBL, and NR databases (**Supplementary Table 8-9**).

### 3.3 Comparative genomic analyses

**Figure 2:**
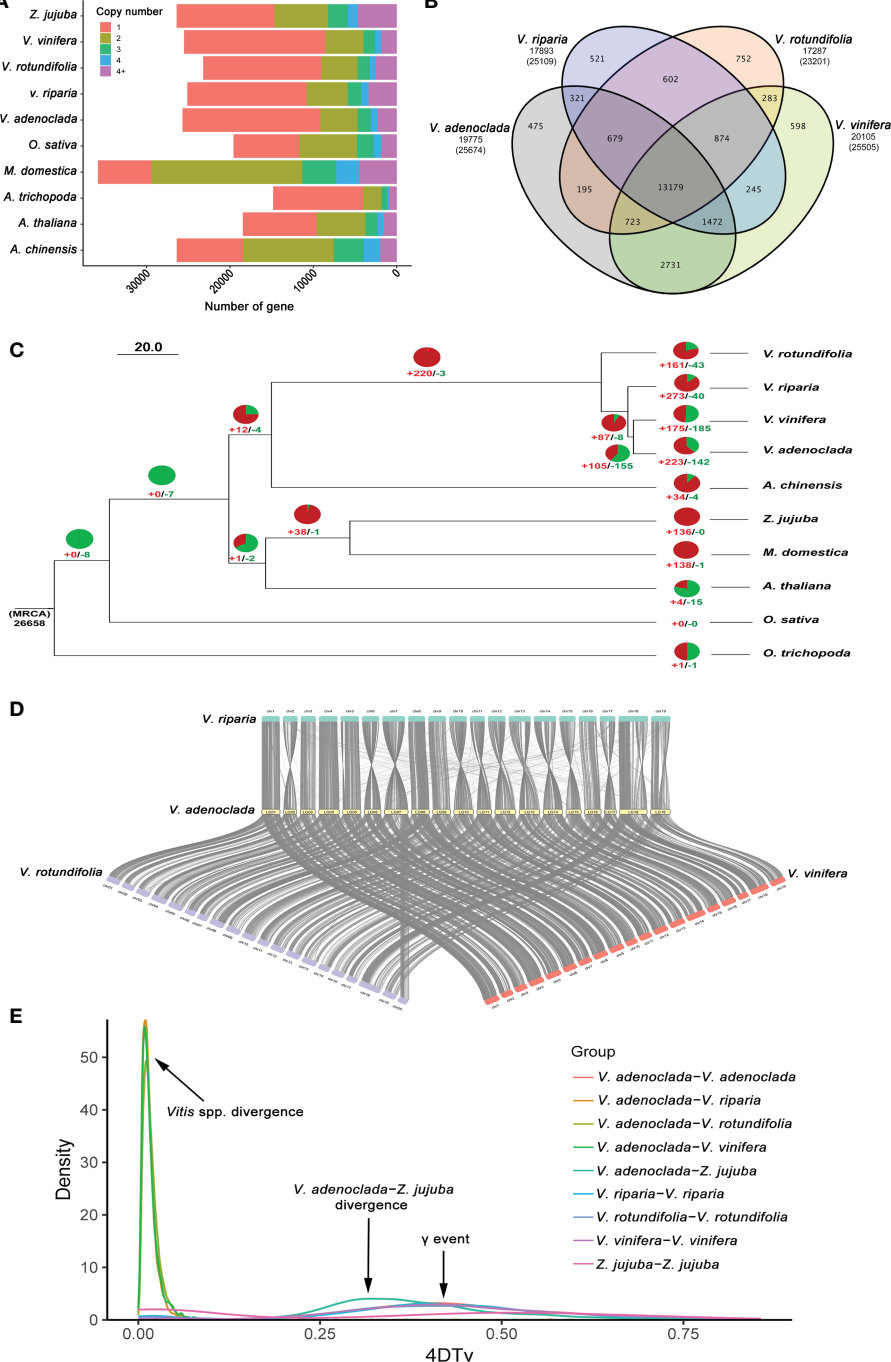
Comparative genomics analysis of *V. adenoclada* and other representative plant species. **(A)** Gene copy number distribution in *V. adenoclada* and nine other plant species. **(B)** Venn diagram of gene families in *V. adenoclada* and the other three grapes. **(C)** Phylogenetic analysis, gene family expansion/contraction analyses and branching time approximations. Green and red represent the number of gene family contraction and expansion occurrences, respectively. Branching times (Mya) are denoted by the numbers adjacent to the nodes. **(D)** Genome collinearity analyses between *V. adenoclada* and the other three grapes of *V. vinifera*, *V. riparia*, and *V. rotundifolia*. **(E)** Distribution of 4DTv among *V. adenoclada*, *V. vinifera*, *V. riparia*, *V. rotundifolia*, and *Z. jujuba* in intra- and intergenomic comparisons.

The genome sequences of nine demonstrative plant species (**Supplementary Table 10**) were selected to perform a gene family cluster analysis with the genome sequence of GH6 (*V. adenoclada*) along with three grapes of PN40024 (*V. vinifera*), Riparia Gloire de Montpellier (*V. riparia*), and Trayshed (*V. rotundifolia*), common jujube (*Ziziphus jujuba*), apple (*Malus domestica*), kiwifruit (*Actinidia chinensis*), another dicots (*Arabidopsis thaliana*), a monocot (*Oryza sativa*), and one in the basal lineage of angiosperms (*Amborella trichopoda*). All genes from 10 selected plant species were clustered into 37,190 gene families. In GH6, a total of 19,775 gene families were identified, 178 of which (comprising 421 genes) were unique to the GH6 genome (**Supplementary Figure 3**). Moreover, in GH6, a total of 16,507 single-copy genes accounted for 57.6 percent of the predicted genes, equal to PN40024 (16,945/56.7%) but higher than Riparia Gloire de Montpellier (14,281/54.8%) and Trayshed (14,152/55.1%) (**Figure 2A**). The clustering of gene families in the four grapes indicated that GH6 harbors 475 specific gene families compared with the other three grapes (**Figure 2B**).

From the ten species, 417 conserved single-copy orthologs were identified and utilized to generate a phylogenetic tree with *A. trichopoda* as an outgroup. According to phylogenetic analysis, *V. adenoclada* is closely connected to *V. vinifera* and forms a clade with *V. riparia* and V*. rotundifolia*. Among four grapes, *V. rotundifolia* of *Muscadinia* and the other grapes of *Euvitis* diverged from their common ancestor at approximately 8.02–24.68 Mya. *V. riparia* of the North America population diverged at approximately 5.15–11.44 Mya before *V. adenoclada* of the East Asian population, which diverged approximately 4.26–9.01 Mya with *V. vinifera* of the European population (**Figure 2C**). These outcomes were consistent with those of prior researches (Liang et al., 2019). Based on the phylogenetic tree, 142 and 223 gene families were contracted and expanded in *V. adenoclada*, respectively (**Figure 2C**). GO functional analysis revealed that the contracted gene families of *V. adenoclada* were involved in lignin catabolic process, apoplast, ADP binding, etc., whereas the expanded gene families of *V. adenoclada* were involved in DNA integration, extracellular region, and ADP binding, etc. (**Supplementary Figure 4**). The gene families of expansion and contraction in the *V. adenoclada* genome relative to their most recent common ancestor (MRCA) are annotated in **Supplementary Table 11-12**.

Genome collinearity analyses between *V. adenoclada* and the other three grapes of *V. vinifera*, *V. riparia*, and *V. rotundifolia* are illustrated (**Figure 2D**). The findings suggested a high degree of gene order and locus conservation between *V. adenoclada* and other three grapes, and chromosome 7 of *V. adenoclada* was observed to be divided into chromosomes 7 and 20 in *V. rotundifolia*, similar as previously reported (Cochetel et al., 2021; Park et al., 2022). Five genomes of *V. adenoclada*, *V. vinifera*, *V. riparia*, *V. rotundifolia*, and *Z. jujuba* were used to calculate distribution of four-fold synonymous third-codon transversion (4DTv) rate (**Figure 2E**). The results showed that all four grapes underwent ancient whole-genome triplication (γ event) in all core eudicots of ~120 Mya (Jiao et al., 2011) before *V. adenoclada* diverged from *Z. jujuba*, whereas there were no recent whole-genome duplication (WGD) events that occurred in the genomes of the four grapes. The 4DTv rate distribution among the species suggested that *Vitis* spp. didn’t occur divergence until very recent age.

### 3.4 Metabolic profiling differences

**Figure 3:**
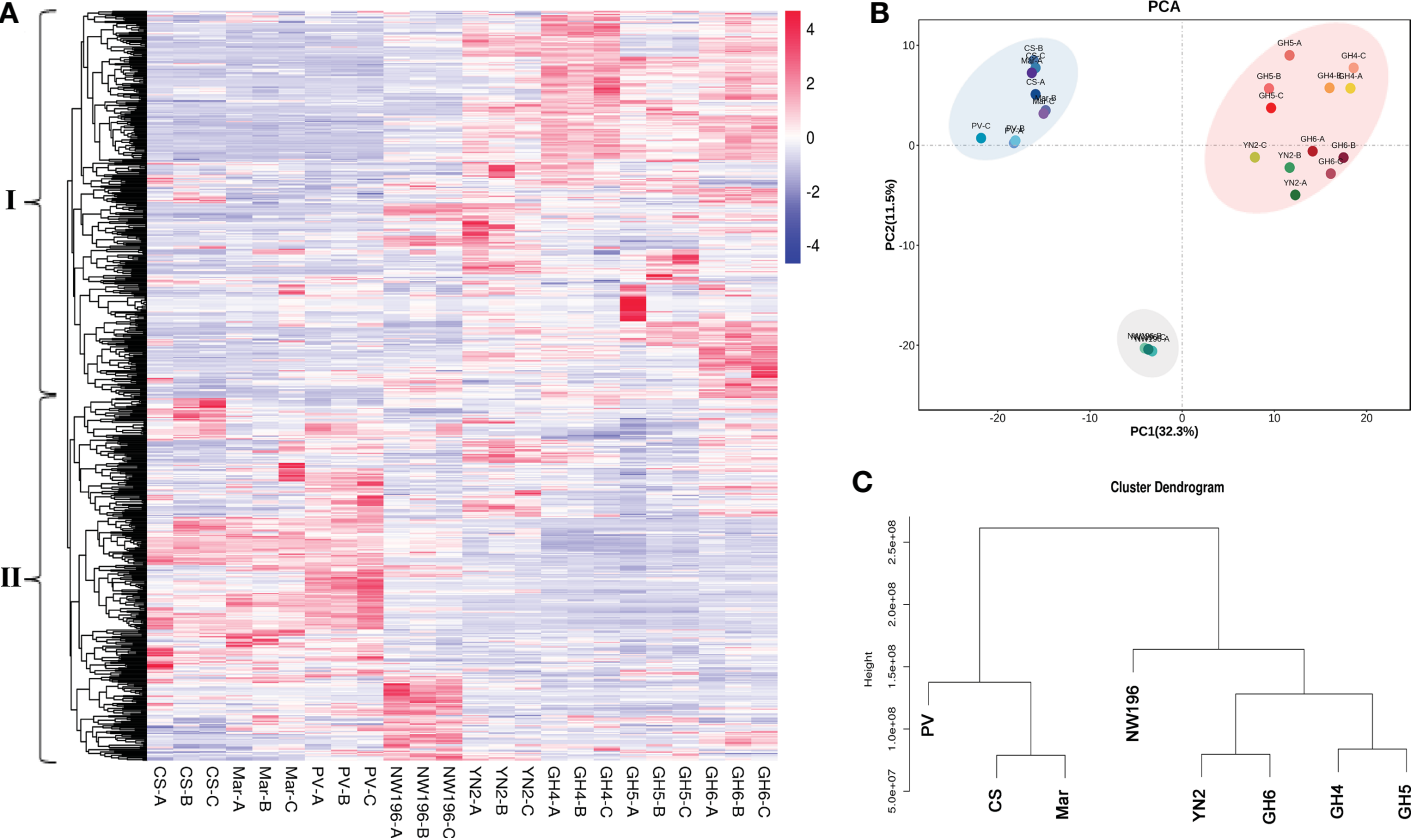
Widely targeted metabolomic analysis in eight grape varieties. **(A)** Overview of 674 metabolites in eight grape varieties containing biological duplications. **(B)** Principal component analysis (PCA). **(C)** Cluster dendrogram of metabolome data from eight grape varieties.

Metabolic profiling was done to identify the metabolite characteristics, especially the quality related products in *V. adenoclada* varieties. Fundamental physical and chemical indexes of the fruit are listed in **Supplementary Table 13**. Widely-targeted metabolomic analyses revealed 674 metabolites, which included organic acids, phenolic acids, tannins, flavonoids, and terpenes (**Supplementary Table 14**). A comparison of the results for all metabolites among the different varieties are presented (**Figure 3A**). These metabolites were divided into two groups by horizontal clustering. Group I included some amino acids and derivatives, most organic acids and phenolic acids, and most flavonols, which accumulated preferentially in *V. adenoclada* varieties. Group II included various anthocyanins, flavanols, tannins, and some lipids, which accumulate more in *V. vinifera* varieties.

PCA divided all eight varieties into three groups and the result was consistent with the cluster dendrogram (**Figures 3B, C**). The results indicated that the four *V. adenoclada* varieties may be grouped into one class and GH6 is much closer to YN2 compared with GH4 and GH5. Although NW196, is closer to *V. adenoclada* varieties compared with the three *V. vinifera* varieties, its metabolic profile showed different characteristics.

### 3.5 Differential accumulation of phenolic compounds among eight grape varieties

**Figure 4:**
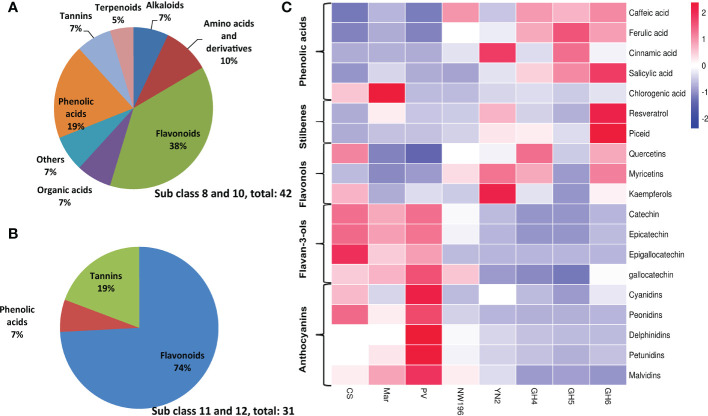
Differential accumulation of phenolic compounds in eight grape varieties. **(A)** The proportion of metabolites in subclasses 8 and 10. **(B)** The proportion of metabolites in subclasses 11 and 12. **(C)** The comparison of non-flavonoids and flavonols in different grape varieties.

To more clearly analyze the metabolite accumulation characteristics of *V. adenoclada*, especially the sequenced variety of GH6, K-means clustering analysis was done. The 674 metabolites in the eight varieties were clustered into 12 subclasses based on metabolic profiling differences (**Supplementary Figure 5**). A total of 42 metabolites in subclasses 8 and 10 exhibited a higher abundance in *V. adenoclada* varieties, hybrid of *V. heyneana* and *V. vinifera* than those in *V. vinifera* varieties (**Supplementary Table 15**), whereas the content of 31 metabolites in subclasses 11 and 12 were higher in the *V. vinifera* varieties (**Supplementary Table 16**). Specifically, in subclasses 8 and 10, flavonoids accounted for 38%, followed by phenolic acids at 19% (**Figure 4A**). Flavonoids also exhibited the highest proportion in subclasses 11 and 12, followed by tannins, and phenolic acids (**Figure 4B**). Most phenolic acids were more abundant in *V. adenoclada* varieties, except chlorogenic acid (**Figure 4C**). The cinnamic acid content in YN2 was higher compared with that in other varieties, whereas the ferulic acid and salicylic acid content in GH5 and GH6 were higher, respectively. The resveratrol and piceid content in GH6 were the highest among all varieties. In general, the content of the three flavonols, quercetins, myricetins, and kaempferols in GH4 and GH6 were higher, followed by CS and NW196. For *V. adenoclada* varieties, the stilbene and flavonol content in GH5 was lower compared with that in GH4 and GH6. For the flavan-3-ols and anthocyanins, the content in *V. vinifera* varieties was higher compared with that in the other varieties, and PV was the highest.

### 3.6 Transcriptome sequencing, clustering, and enrichment analysis of degs in phenolic synthesis pathway

After alignment with the self-testing genome, we obtained 35.77-51.55 million total reads (**Supplementary Table 17**). The matching rate of these high-quality reads to the reference genome ranged from 84.05% to 92.50%. The GC content of the 24 samples ranged from 45.57% to 46.61%. The Q30 percentage of them was ≥93.46%, suggesting that the sequencing data was reliable and satisfied the threshold for downstream analysis.

**Figure 5:**
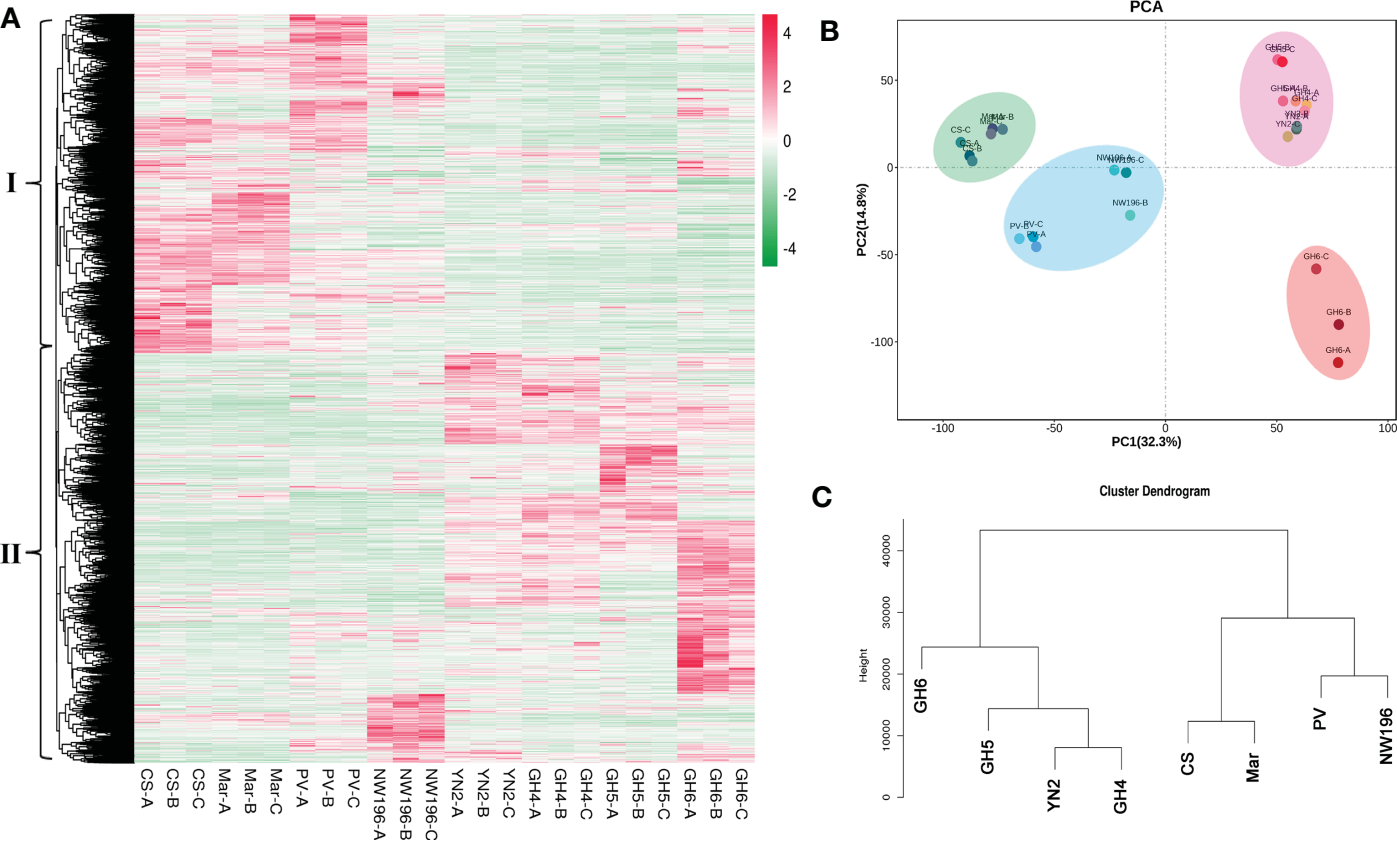
Transcriptome data analysis in eight grape varieties. **(A)** Overview of 30,257 genes in eight grape varieties containing biological duplications. **(B)** Principal component analysis (PCA). **(C)** Cluster dendrogram of transcriptome data from grape varieties.

In total, 30,257 genes were found to be expressed in 24 grape samples, which contained 1,597 novel genes. In addition, a total of 13,423 DEGs were identified *via* DESeq2 centered on |log2fold-change| ≥1 and a false discovery rate (FDR) < 0.05 in all samples (**Supplementary Table 18**). Based on the expression pattern among the different grape varieties, these DEGs were divided into two groups (**Figure 5A**). We used R language pheatmap package to calculate the euclidean distance between two samples by using expression information. The clustering in **Figure 5A** was achieved by euclidean distance. Group I genes were more abundant in *V. vinifera* varieties, whereas group II genes were more abundant in *V. adenoclada* varieties, a hybrid of *V. heyneana* and *V. vinifera*. The PCA and cluster dendrogram of the transcriptome were basically congruent with the results of the gene expression pattern classification (**Figures 5B, C**).

**Figure 6:**
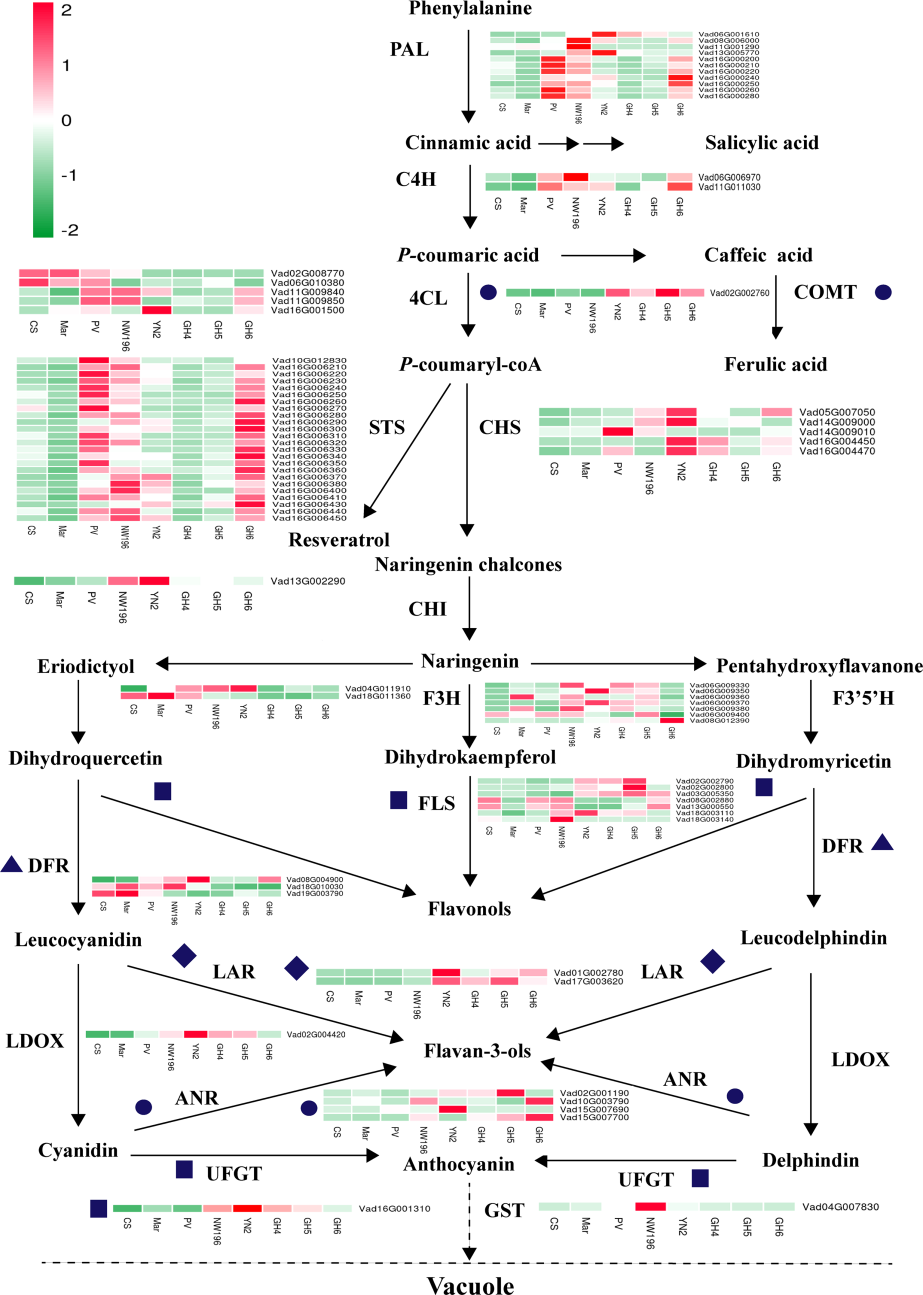
Transcriptomic analysis of structural DEGs implicated in phenolic biosynthesis within berries of eight grape varieties. 4CL, 4-coumarate: CoA ligase; ANR, anthocyanidin reductase; C4H, *trans*-cinnamate 4-monooxygenase; CHS, chalcone synthase; CHI, chalcone isomerase; COMT, caffeic acid 3-*O*-methyltransferase; DFR, dihydroflavonol reductase; F3H, flavonoid 3-hydroxylase; F3’5’H, flavonoid 3’,5’-hydroxylase; FLS, flavonol synthase; GST, glutathione S-transferase; LAR, leucoanthocyanidin reductase; LDOX, leucoanthocyanidin dioxygenase; UFGT, UDP-glucose: flavonoid 3-*O*-glucosyltransferase; PAL, phenylalanine ammonia-lyase; STS, stilbene synthase. As highlighted in the legend, each square in the heatmap next to its gene names corresponds to the mean FPKM value of the gene in each sample.

We employed RNA-seq to assess variations in gene expression at the transcript level in the berries of eight grape varieties in relation to the phenylpropanoid/flavonoid biosynthesis pathway-related structural genes (**Figure 6**). The findings suggested that the transcription of genes encoding enzymes upstream of the phenylpropane metabolic pathway, including *PAL* (EC:4.3.1.24), *C4H* (EC:1.14.13.11), and *STS* (EC 2.3.1.74), showed significantly higher expressions in berries of PV, NW196, YN2, and GH6. Expression of the *COMT* (EC: 2.1.1.68) gene in four *V. adenoclada* varieties higher than the other four varieties, whereas most of *4CL* (EC 6.2.1.12) presented significantly or moderately higher expressions in three *V. vinifera* varieties or NW196. Generally, *V. adenoclada* varieties exhibited many similarities when compared with the varieties that have *V. vinifera* consanguinity. However, there were also some differences between the four *V. adenoclada* varieties. Specifically, *CHS* (EC 2.3.1.74), *CHI* (CHI, EC 5.5.1.6), and *F3H* (EC:1.14.11.9) exhibited higher levels of expression in YN2. In general, almost all members of *FLS*, *LAR* (EC 1.17.1.3), *ANR* (EC 1.3.1.77), and *UFGT* (EC 2.4.1.115) presented higher expressions in four *V. adenoclada* varieties compared with the *V. vinifera* varieties. In addition, the gene encoding glutathione S-transferase (GST4, Vad04G007830) showed the highest expression in NW196.

### 3.7 Co-expression analysis between phenolic biosynthetic pathway genes and transcription factor (TF) genes

**Figure 7:**
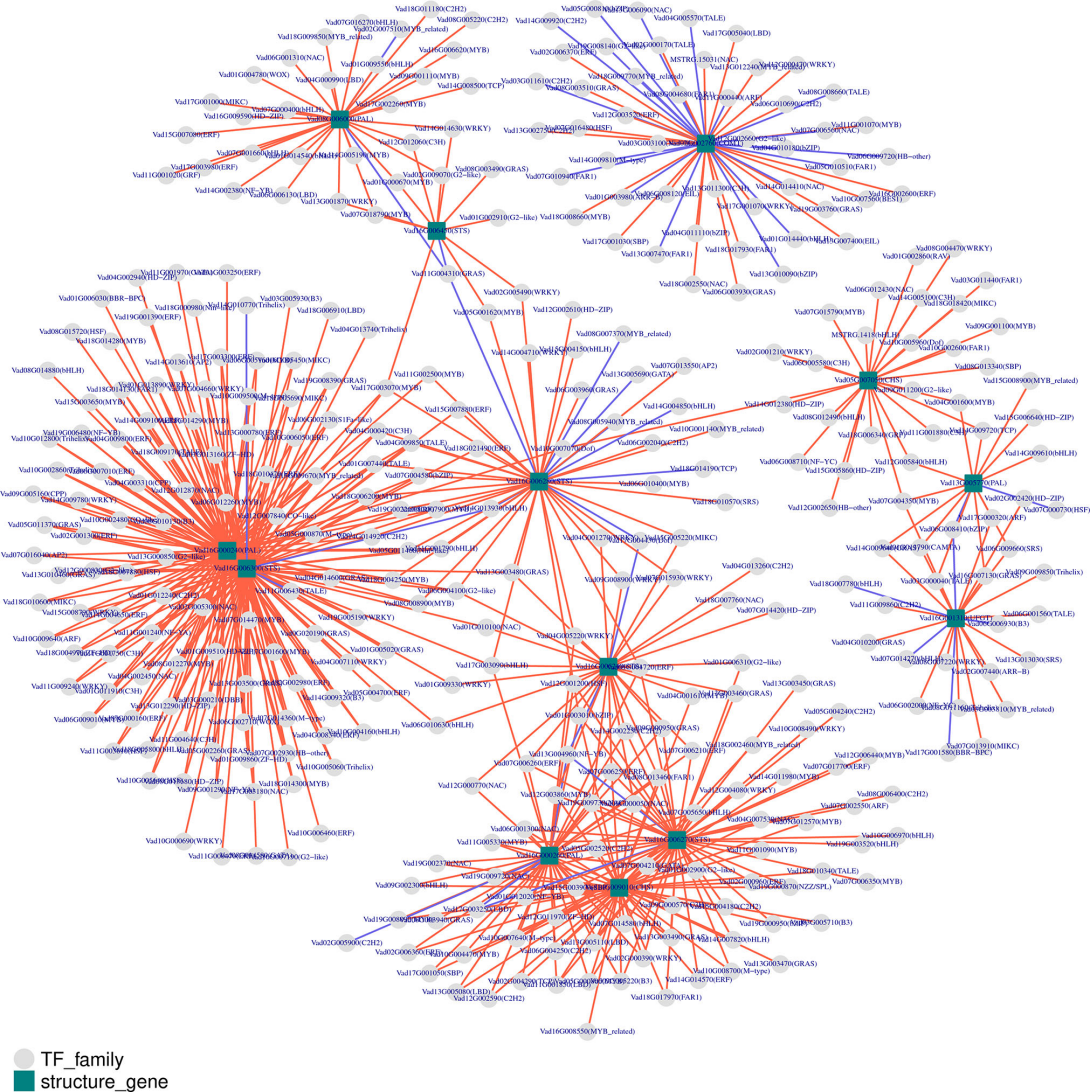
Co-expression analysis of key enzymes located in phenolic biosynthesis and transcription factors (TFs) in berries from eight grape varieties. Key enzymes are shown in rectangles and TFs in circles. CHS, chalcone synthase; COMT, caffeic acid 3-*O*-methyltransferase; PAL, phenylalanine ammonia-lyase; STS, Stilbene synthase; UFGT, UDP-glucose: flavonoid 3-*O*-glucosyltransferase. Positive and negative correlations are provided by red arrows and purple lines, respectively.

To analyze the expression of TFs related to phenolic metabolism, the predicted protein sequences were compared using the plant TF database by hmmscan (**Figure 7**). A total of 1442 TFs were predicted, and the top four transcription factors were MYB (130), bHLH (121), ERF (104), and C2H2 (91) (**Supplementary Table 19**). To predict the TFs and genes with high connectivity that regulate key structural genes in the phenolic biosynthetic pathway, a co-expression analysis was done between the structural and TF genes. Five phenolic synthesis genes, including genes encoding PALs, COMT, STSs, CHSs, and UFGT, were selected as “target genes.” The absolute value of the Pearson Correlation Coefficient and *p*-value between them and the expression levels of TF genes using RNA-seq data was calculated. We selected an absolute value for PPC greater than 0.85 and a *p*-value less than 0.01 as conditions. For structural genes with multiple transcripts, we selected the most representative ones through expression pattern clustering for analysis.

Among the identified co-expressed TFs, the most abundant positively correlated TFs were members of the MYB, ERF, bHLH, WRKY, GRAS, NAC, and C2H2 families. For phenylpropane metabolic pathway entry enzymes, four members (Vad08G006000, Vad13G005770, Vad16G000240 and Vad16G000260) of the PAL family were mainly positively regulated by MYB, ERF, bHLH, GRAS, NAC, WRKY and C2H2 families. On the other hand, a total of 218 TFs regulated the five members (Vad16G006240, Vad16G006270, Vad16G006280, Vad16G006300, Vad16G006450) of the STSs, mainly belonging to MYB, ERF, bHLH, WRKY, GRAS and NAC families. Among them, 16 TFs played a negative regulatory role. In addition, STS (Vad16G006300) was in the network with PAL (Vad16G000240), and they were regulated by 102 TFs. Of note, TALE (Vad11G006430) was the only negatively regulated TF among the 102 common TFs. However, STS (Vad16G006450) and PAL (Vad08G006000) were linked by only five TFs: GRAS (Vad11G004310), C3H (Vad12G012060), G2-like (Vad02G009070), MYB (Vad01G000670) and WRKY (Vad13G001870). In addition, GRAS (Vad11G004310) was the only negatively regulated TF. In the other network, PAL (Vad16G000260), STS (Vad16G006270) and CHS (Vad14G009010) had 16 common TFs. COMT (Vad02G002760) alone formed a network as a “target gene” with 48 TFs, and 21 of them exercised negative regulation. Moreover, 24 TFs had regulation effect on UFGT (Vad16G001310), and B3 (Vad06G006930), BBR-BPC (Vad17G001580), bHLH (Vad18G007780, Vad07G014270) presented negative regulation of it. bZIP (Vad06G008410) had negative regulatory effects on UFGT (Vad16G001310) and PAL (Vad13G005770). Interestingly, MYB (Vad07G004350) was the only TF that connected PAL (Vad13G005770), CHS (Vad05G007050) and UFGT (Vad16G001310) in the same network.

### 3.8 Weighted gene co-expression network analysis (WGCNA) of the DEGs

**Figure 8:**
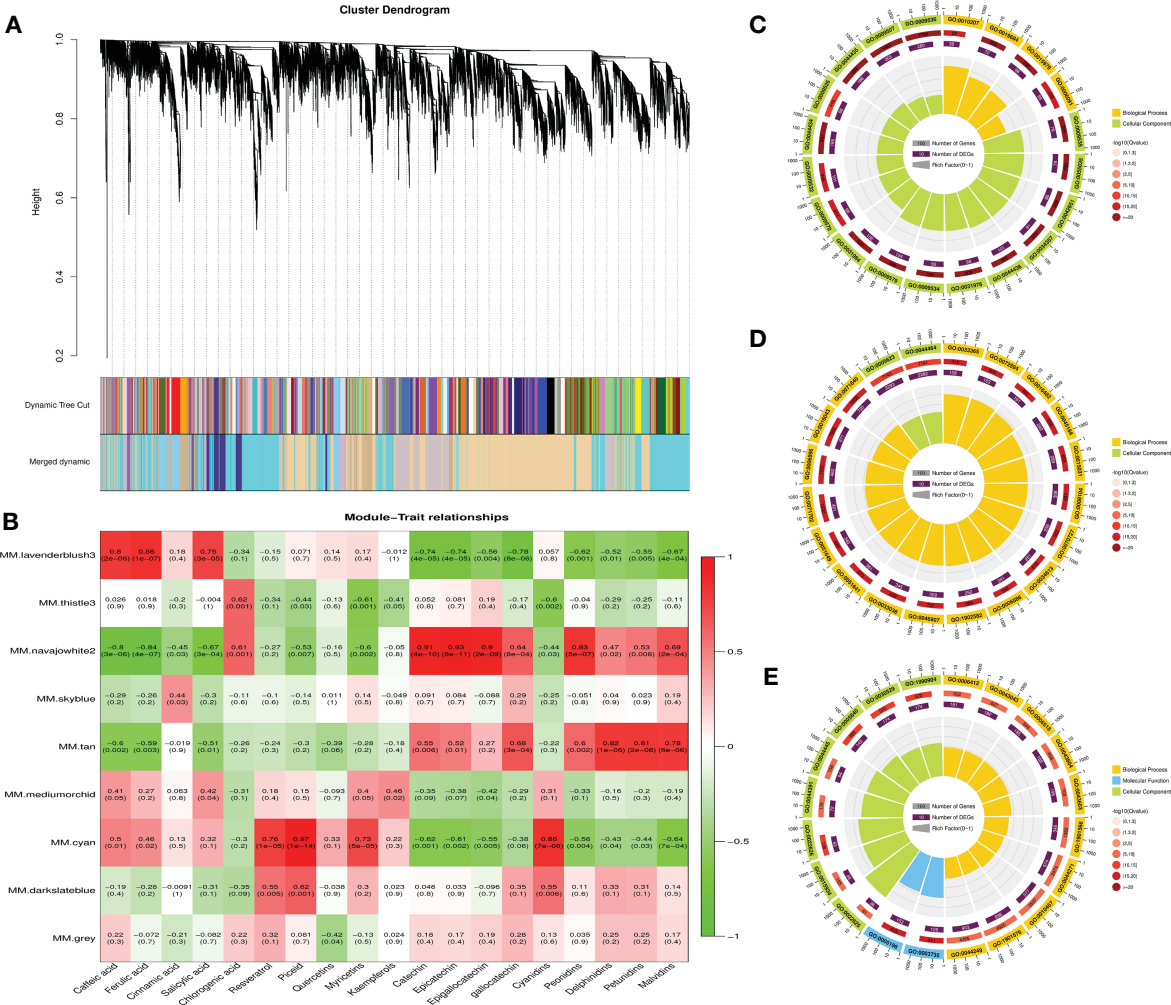
Weighted gene co-expression network analysis (WGCNA) of DEGs among different grape varieties and GO enrichment analyses of DEGs associated with the accumulation of phenolic compounds. **(A)** Cluster dendrogram showing co-expression modules identified by WGCNA. The major tree branches constitute 9 modules labeled by different colors. **(B)** Module–trait relationships. Each row corresponds to a module. Each column corresponds to a group of phenolic compounds. The color and data of each cell indicates the correlation coefficient between the module and the phenolic compound. A high degree of correlation between a specific module and the phenolic compound is indicated by red (positive) or green (negative). Red to green represents a positive to negative correlation between them. **(C)** GO enrichment analysis of DEGs in the lavenderblush module. **(D)** GO enrichment analysis of DEGs in the navajowhite module. **(E)** GO enrichment analysis of DEGs in the cyan module.

To gain further insight into the accumulation and variation of metabolites among the grapes, a WGCNA was performed to identify the co-expression networks of DEGs. Co-expression modules are defined as clusters of highly interconnected genes with high correlation coefficients. Genes with similar expression patterns were clustered into 9 distinct modules with gene numbers ranging from 64 (gray) to 6,952 (navajowhite) (**Figure 8A**). Then, the association between modules and specific phenolic compounds were analyzed (**Figure 8B**). Notably, the lavenderblush module consisted of DEGs that were significantly (p-value ≤ 0.01) positively correlated with caffeic acid, ferulic acid, and salicylic acid. For navajowhite and tan modules, all of the DEGs were significantly positively correlated with flavan-3-ols (catechin, epicatechin, gallocatechin, and epigallocatechin) and most anthocyanins (peonidins, delphinidins, petunidins, and malvidins). However, the cyan module consisted of DEGs that were significantly positively correlated with stilbenes (resveratrol and piceid) and myricetins. Of note, STSs were mainly found in darkslateblue module, and the correlation between different transcripts and TFs deserves further study in the future.

GO enrichment analysis revealed that the top 20 GO terms in the lavenderblush and navajowhite modules all belonged to biological processes and cellular components. GO: 0009536 (Plastid, 1,708 genes) and GO: 0005623 (cell, 7,143 genes) exhibited the largest number of background genes, with 280 and 2,293 DEGs, respectively (**Figures 8C, D**). In addition, the top 20 GO terms in the cyan module all belonged to biological processes, molecular functions, and cellular components. GO: 1901576 (organic substance biosynthetic process, 4,505 genes) exhibited the largest number of background genes with 936 DEGs (**Figure 8E**).

There were 93 TFs annotated in the lavenderblush module, most of which were bHLH (11) and ERF (10) family members, and most connected with the module were SCL13, BBX19, ERF5, GLK1, ARF19, WRKY28, MYB73, ERF012, BLH9, and MYB5 (**Supplementary Table 20**). COMT was also located in this module and the TFs with a correlation coefficient >0.8 were: ERF5 (Vad16G002600), ARF19 (Vad11G000440), MYB2 (Vad11G001070), BLH9 (Vad08G008660), WRKY28 (Vad12G000470), GLK1 (Vad12G002660), MYB73-1 (Vad18G008660), and MYB73-2 (Vad03G006650) (**Supplementary Table 21**). These TFs may be involved in the regulation of ferulic acid biosynthesis.

There were 315 TFs annotated in the cyan module, most of which were bHLH (26) and ERF (27) family members, and most connected with the module were WRKY65, WRKY21, bHLH130, ASIL2, COL10, ARF9, STOP1, ERF118, PIL15, and SCL14 (**Supplementary Table 22**). FLS (Vad18G003110) was located in the cyan module, and the TFs with a correlation coefficient >0.8 were: ARF2A (Vad17G000320), BLH7 (Vad03G000040), HOX16 (Vad14G012380), LRP1 (Vad06G009660), and MYB12 (Vad07G004350). UFGT (Vad16G001310) was also located in the cyan module and the TFs with a correlation coefficient >0.8 were: ZFP2 (Vad11G009860), WRKY44 (Vad08G007220), MYB12 (Vad07G004350), ARF2A (Vad17G000320), HOX22 (Vad02G002420), and LRP1 (Vad06G009660) (**Supplementary Table 23**). Therefore, these TFs may play a regulatory role in the synthesis of flavonols and anthocyanins.

## 4 Discussion

Since the publication of the first grape genome (PN40024), studies on grapes have made a qualitative leap at the molecular level (Jaillon et al., 2007). In recent years, whole-genome sequencing or resequencing of wild *Vitis* species from East Asian populations has been reported, especially those that originate from China (Liang et al., 2019; Wang et al., 2021). This has provided rich data for illuminating the evolutionary biology of the *Vitis* species and has achieved a more accurate comparison with different species/varieties at the genomic level. However, little is known regarding the metabolism and accumulation of the East Asian grapevine using a self-testing genome.

We provide a high-quality, chromosome-level genome assembly of GH6, a *V. adenoclada* varieties, based on a collection of Illumina and ONT sequence data followed by Hi-C mapping. The resultant genome size of GH6 was 498.27 Mb with a lower level of gaps (0.059 Mb) compared with PN40024 (~15 Mb) (GenBank assembly accession: GCA_000003745.2), Trayshed (~1 Mb) (Cochetel et al., 2021), Shanputao (~4 Mb) (Wang et al., 2021), Riparia Gloire de Montpellier (~6 Mb) (GenBank assembly accession: GCA_004353265.1). A total of 481.44 Mb sequences were anchored to 19 chromosomes of GH6 *via* the Hi-C assembly, accounting for 96.62% of the final genome assembly. This rate was higher compared with that of PN40024 (87.7%) (GenBank assembly accession: GCA_000003745.2), Shanputao (82.6%) (Wang et al., 2021), Trayshed (92.5%) (Cochetel et al., 2021) and Riparia Gloire de Montpellier (94.2%) (GenBank assembly accession: GCA_004353265.1). The high-quality and chromosome-level genome of GH6 that was deciphered will be helpful for the utilization of the East Asian wild germplasm resources of *Vitis* spp. for future grape genetic improvement and evolutionary studies. We also found that quite a number of gene family expansions in the genome of GH6 associated with defense response including RPP13-like proteins, TMV resistance proteins, RPM1s, STSs, and so on, which suggests that the expanded gene families of GH6 may contribute to resistance against diseases.

Chinese wild grapes generally have the advantages of high yield, strong resistance to stress, and a rich content of phenolics. The most studied and reported species include *V. amurensis*, *Vitis pseudoreticulata*, *V. heyneana* and *V. davidii* (Ma et al., 2018; Ju et al., 2020; Wang et al., 2021; Yan et al., 2021). In the current work, phenols are crucial in differentiating species and varieties using a metabolomics approach. Most phenolic acids were found in higher concentrations in the fruits of *V. adenoclada* and the hybrid with East Asian lineage than in *V. vinifera*. Phenolic acids are non-flavonoid phenolic compounds synthesized by the phenylpropane metabolic pathway (Garrido and Borges, 2013). Here, we determined that the content of caffeic acid, cinnamic acid, ferulic acid, and salicylic acid in *V. adenoclada* berries were higher compared with those of *V. vinifera*.

Resveratrol plays an important role in resistance to biotic and abiotic stresses, especially in pathogen resistance (Yan et al., 2021). In the current investigation, among all varieties, we observed that GH6 has the highest concentration of resveratrol and its glycosides (piceid). It should be noted that wine grapes cultivated in the south subtropical region are more likely to suffer from the threat of pathogens owing to hot and humid condition, which has a devastating impact on the quality and yield (Bai et al., 2008; Liu K. Y., 2012). Therefore, effective utilization of the *V. adenoclada* grape will facilitate the breeding of new grapevine cultivars during the breeding process.

Flavonoids in grapes mainly include flavonols, flavanols, and anthocyanins (Wu et al., 2020). In this study, the flavonol content in GH4 and GH6 was the highest among all varieties. However, the flavan-3-ols and anthocyanins of *V. vinifera* varieties were most abundant, and NW196 contained higher levels than *V. adenoclada* varieties. The previous study demonstrated that the content of flavonoids in *V. heyneana* and *V. davidii* was higher compared with that of CS, whereas *V. adenoclada* was similar (Jiang, 2016). From the trend analysis, the higher content of metabolites in European or East Asian grape species were associated with flavonoids. Therefore, it is necessary to further explore the phenolics synthesis in different grape varieties by RNA-seq.

Currently, studies on the characteristics and regulation of grape fruit quality revealed by metabolomics and transcriptomics have been primarily focused on *V. vinifera* (Sun et al., 2019; Yang et al., 2020). For unpublished species and variety genomes, most transcriptome studies have selected the genome assembly of PN40024 as a reference genome (Cheng et al., 2019; Zhang et al., 2020). In the present study, the mapped rates of *V. adenoclada* varieties using a self-testing genome increased 0.86%–1.46% compared with that of the PN40024 genome (**Supplementary Table 24**). In contrast, the mapped rates of *V. vinifera* varieties and NW196 using the genome of GH6 decreased by 6.35%–8.84%. This indicates that selecting genomes with a closer genetic background as a reference can reduce error and improve the accuracy of the comparison.

In the present study, the *V. adenoclada* varieties were clearly distinguished from the *V. vinifera* varieties and NW196. There were significant variations in the expression of 15 structural genes in the phenylpropane metabolic pathway across the eight varieties, ranging from early *PAL* to late *GST*. PAL as an entry enzyme in the phenylpropane pathway (Sun et al., 2019). In this study, most PAL members were highly expressed in PV, whereas they were highly expressed in GH6 among the *V. adenoclada* varieties. In addition, the *C4H* and *COMT* were closely related to phenolic acid synthesis, whereas 4CL catalyzes *p*-coumaric acid and continues to lead the flavonoid synthesis pathway (Cheng et al., 2019). The expression of C4H was higher in PV, NW196, and GH6, whereas those of COMT and 4CL were higher in *V. adenoclada* and *V. vinifera* varieties, respectively. This could explain why the phenolic acid concentration of *V. adenoclada* berries was higher than that of *V. vinifera* berries. STS is a key enzyme involved in the biosynthesis of resveratrol and has been linked to plant resistance to fungal diseases (Shi et al., 2014). In the current study, STSs exhibited higher expression level in GH6, which may explain the high content of resveratrol and piceid in the results of the widely-targeted metabolome. From our previous analysis, the content of flavanols and anthocyanins in PV was the highest among the eight grape varieties. However, *CHS*, *LAR*, *ANR*, *UFGT*, and *GST4* were generally highly expressed in *V. adenoclada* varieties or NW196. These results indicate that the synthesis of flavonoids was more affected by the upstream genes, *PAL* or *4CL*. The previous study regarding the anthocyanins in *V. vinifera* and the hybrid of *V. vinifera* and *V. amurensis* arrived at similar estimates (Liu et al., 2021).

To better understand the regulatory process of phenolic metabolism in different grape varieties, we performed a TF prediction and genome-wide co-expression network analysis. Previous studies on the regulation of transcription factors on flavonoid pathway genes have primarily focused on *V. vinifera*, but there have been few reports on the wild resources of the East Asian species (Pilati et al., 2007; Terrier et al., 2009; Sun et al., 2019; Cheng et al., 2021). In the present study, a total of 1,442 transcription factors were predicted and the MYB family was the most abundant. To date, multiple TFs belonging to the MYB, WRKY, ERF, and bHLH families have been shown to regulate flavonoid biosynthesis, and the MYB family is the most well-studied (Wei et al., 2021). Many key MYB TFs, including MYBA1, MYBA2, MYB5A, MYB5B, MYBPA1 MYBPA2, MYB4, and MYB86 have been identified that promote or inhibit flavonoid biosynthesis in the grapevine (Sun et al., 2019; Cheng et al., 2021). From our study, these TFs not only regulate flavonoid synthesis, but also play an important role in the synthesis of non-flavonoid phenols. Co-expression analysis revealed that the PALs and STSs family members were positively regulated by MYB, ERF, bHLH, GRAS, NAC and WRKY family TFs. Previous studies have demonstrated that *MYBF1* (also known as *MYB12*) was specifically responsible for flavonol biosynthesis in grapes (Sun et al., 2019). In our study, MYB12 (Vad07G004350) positively regulated PAL (Vad13G005770), CHS (Vad05G007050), FLS (Vad18G003110) and UFGT (Vad16G001310). WGCNA analysis further indicated that COMT is highly correlated with the MYB family genes, such as MYB2 (Vad11G001070), MYB73-1 (Vad18G008660), and MYB73-2 (Vad03G006650). Presumably, these TFs are involved in the regulation of ferulic acid biosynthesis. Target genes of *MYBPA1*, *MYBPA2*, and *MYBPAR* in the grape are *LAR* and *ANR*, which regulate the synthesis of proanthocyanidins (Bogs et al., 2007; Terrier et al., 2009; Koyama et al., 2014). In the present study, MYB5b (Vad06G000560) and MYBPAR (Vad11G001070) were highly expressed in four *V. adenoclada* varieties, which corresponded with the higher expressions of LAR and ANR in these varieties. However, MYBPA1 (Vad15G006940) was highly expressed in CS and Mar. In recent years, MYB4 and MYBC2-L1 have been shown to inhibit proanthocyanidin and anthocyanin synthesis (Cavallini et al., 2015; Pérez-Díaz et al., 2016; Zhu et al., 2019). From the results of our study, MYB4 (Vad05G005030) and MYBC2-L1 (Vad01G004530) were highly expressed in YN2 and GH6. This may inhibit the synthesis of flavan-3-ols and anthocyanins in both varieties to some extent. From the present study, transcriptome analysis with self-testing genome will provide insight into the synthesis and regulation of phenols in *V. adenoclada* grapes.

## Data availability statement

The datasets presented in this study can be found in online repositories. The names of the repository/repositories and accession number(s) can be found below: https://ngdc.cncb.ac.cn/, PRJCA009537, https://ngdc.cncb.ac.cn/, PRJCA009575.

## Author contributions

GC, DW, RG and SZ conceptualized the study and contributed to original draft and funding acquisition. GC and SZ contributed to reviewing and editing of the manuscript. HL, RW, HY and LL participated in experimental processing and collecting material. GC, DW, ZW, XM and NY contributed to resources. GC, JZ, LX and SZ analyzed the data. SZ supervised the project. All authors contributed to the article and approved the submitted version.
