# Supplementary material for: Chromosome-scale genomics, metabolomics, and transcriptomics provide insight into the synthesis and regulation of phenols in Vitis adenoclada grapes
Source: Front Plant Sci. 2023 Jan 25;14:1124046. doi: 10.3389/fpls.2023.1124046 (PMC9907855; doi:10.3389/fpls.2023.1124046)
Supplement: Supplementary file 2 [file DataSheet_1.pdf]

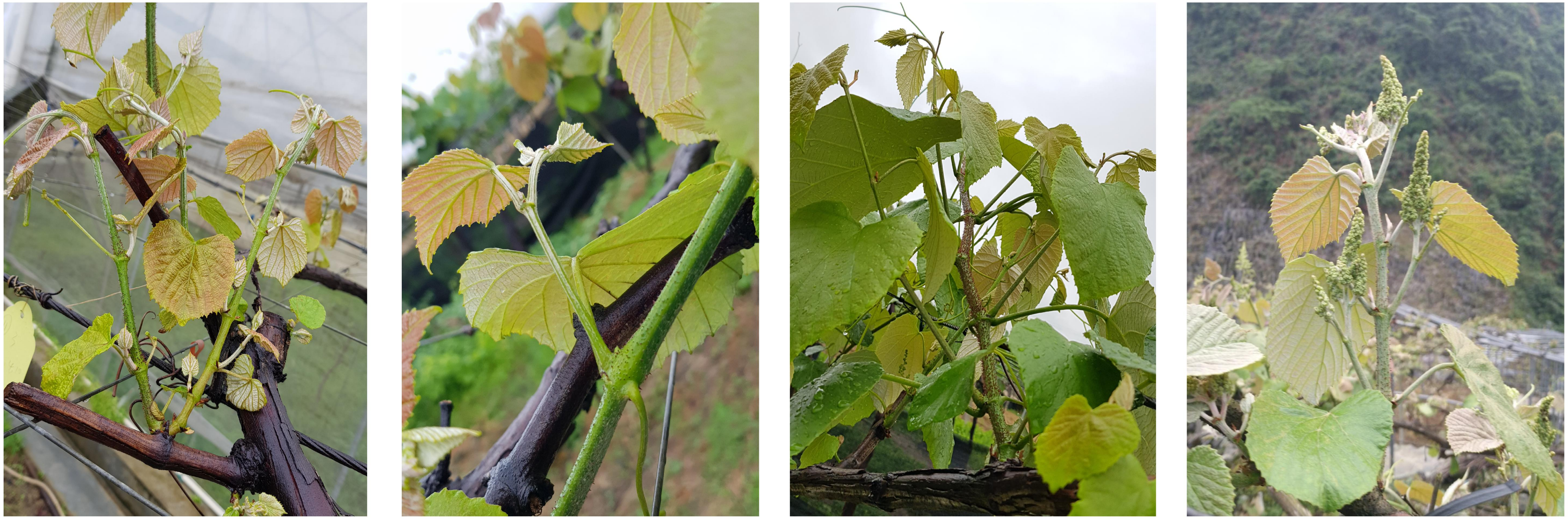

YN2 GH4 GH5 GH6

Supplementary Figure 1. Glandular hair characteristics of four *V. adenoclada* grape varieties

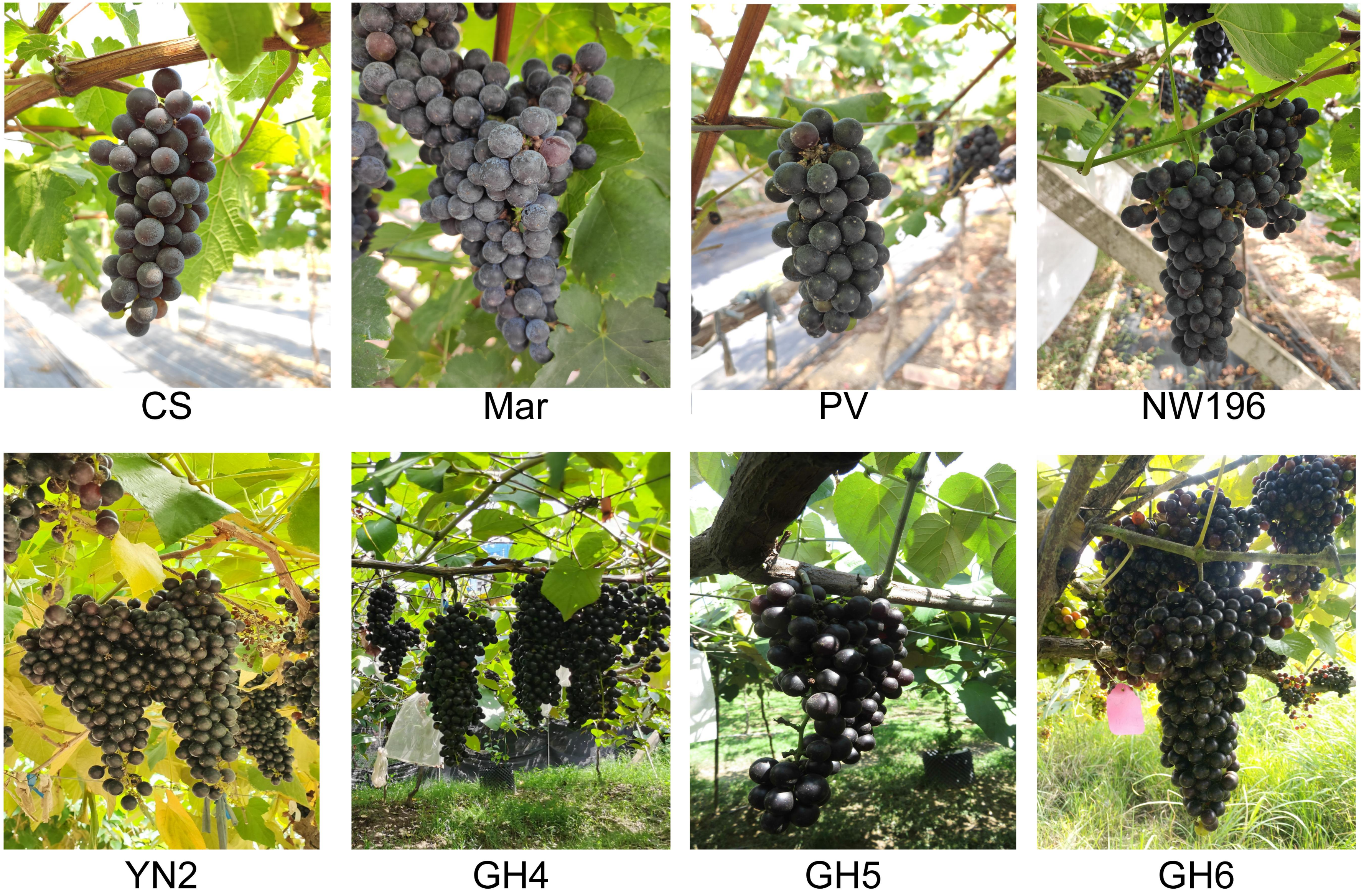

Supplementary Figure 2. Photos of eight grapes at harvest stage

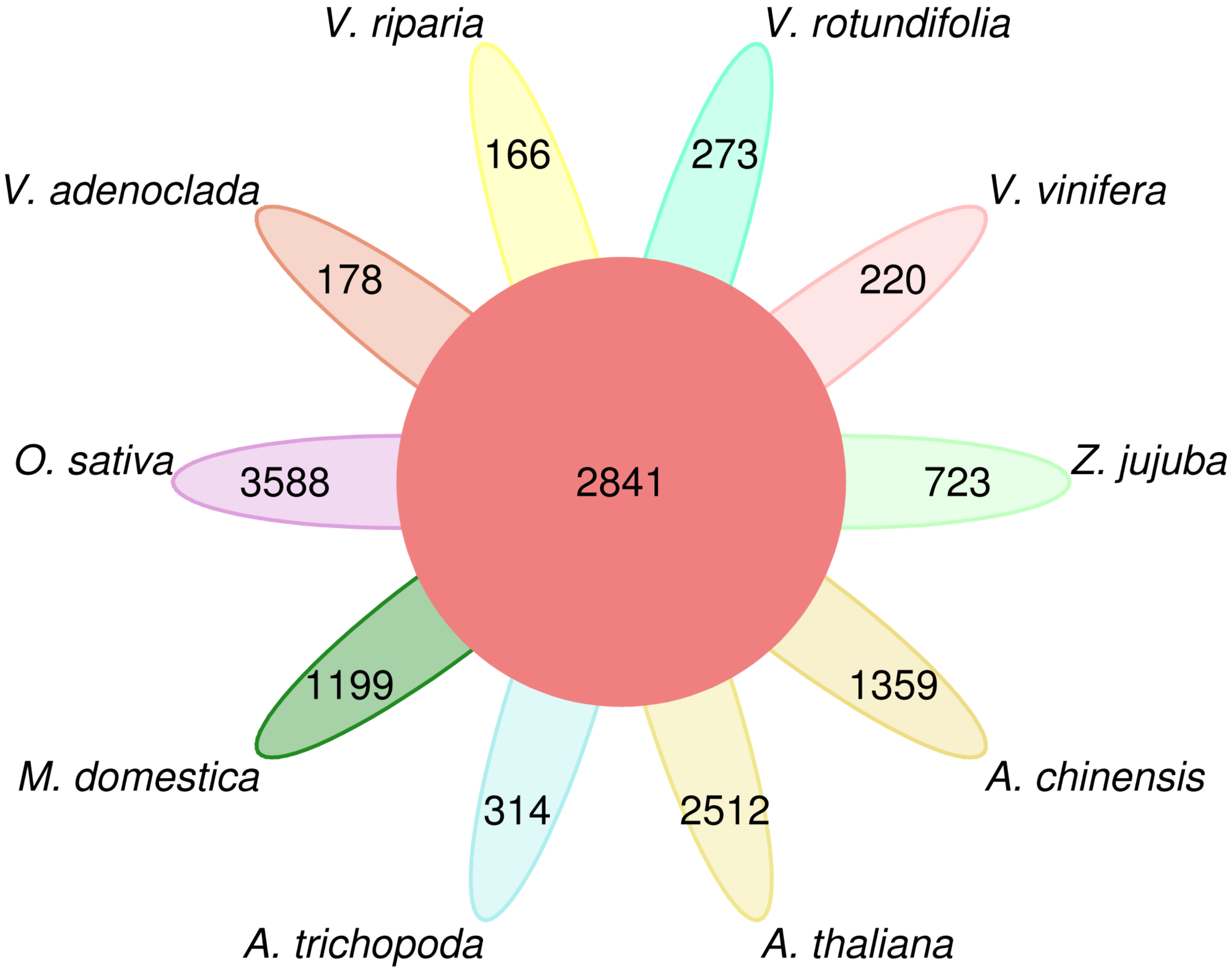

Supplementary Figure 3. The clustering of gene families

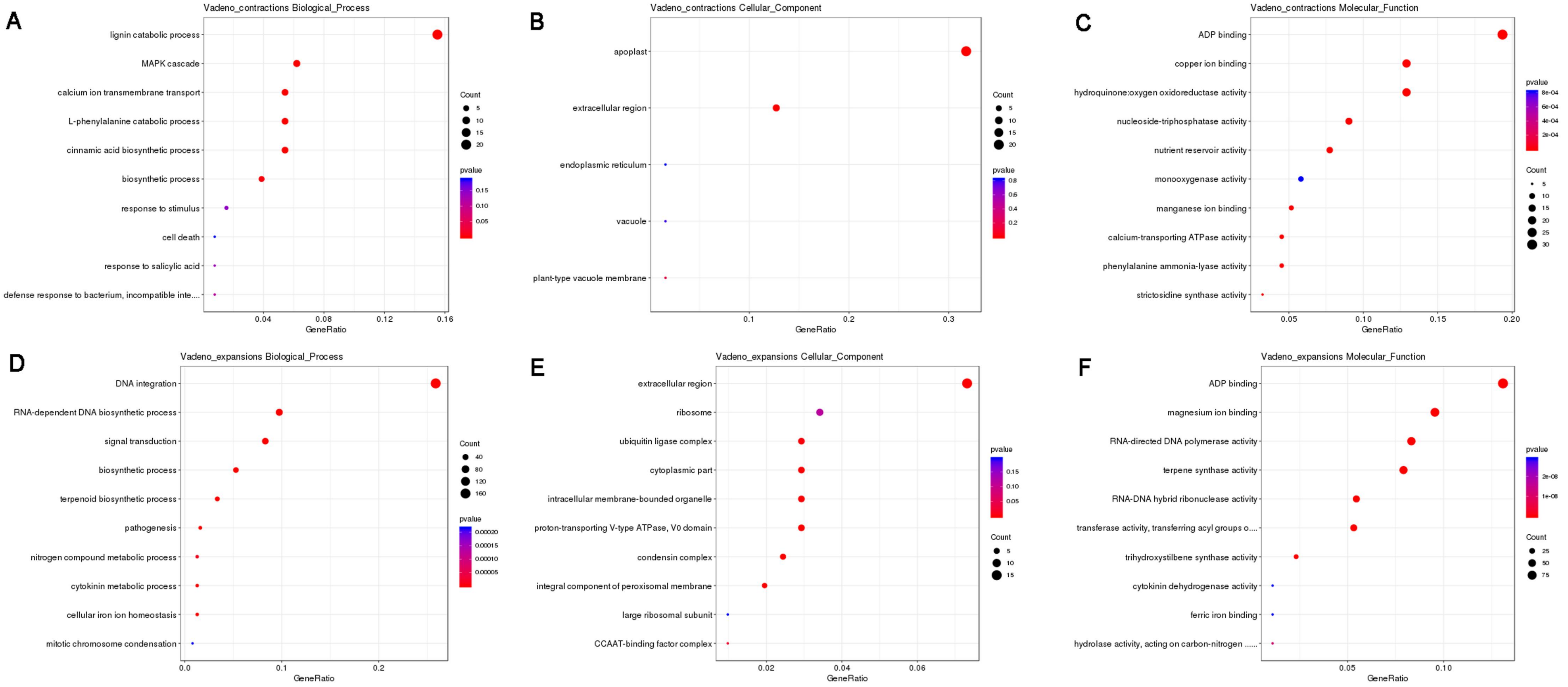

Supplementary Figure 4. GO enrichment analyses of expanded and contracted gene families

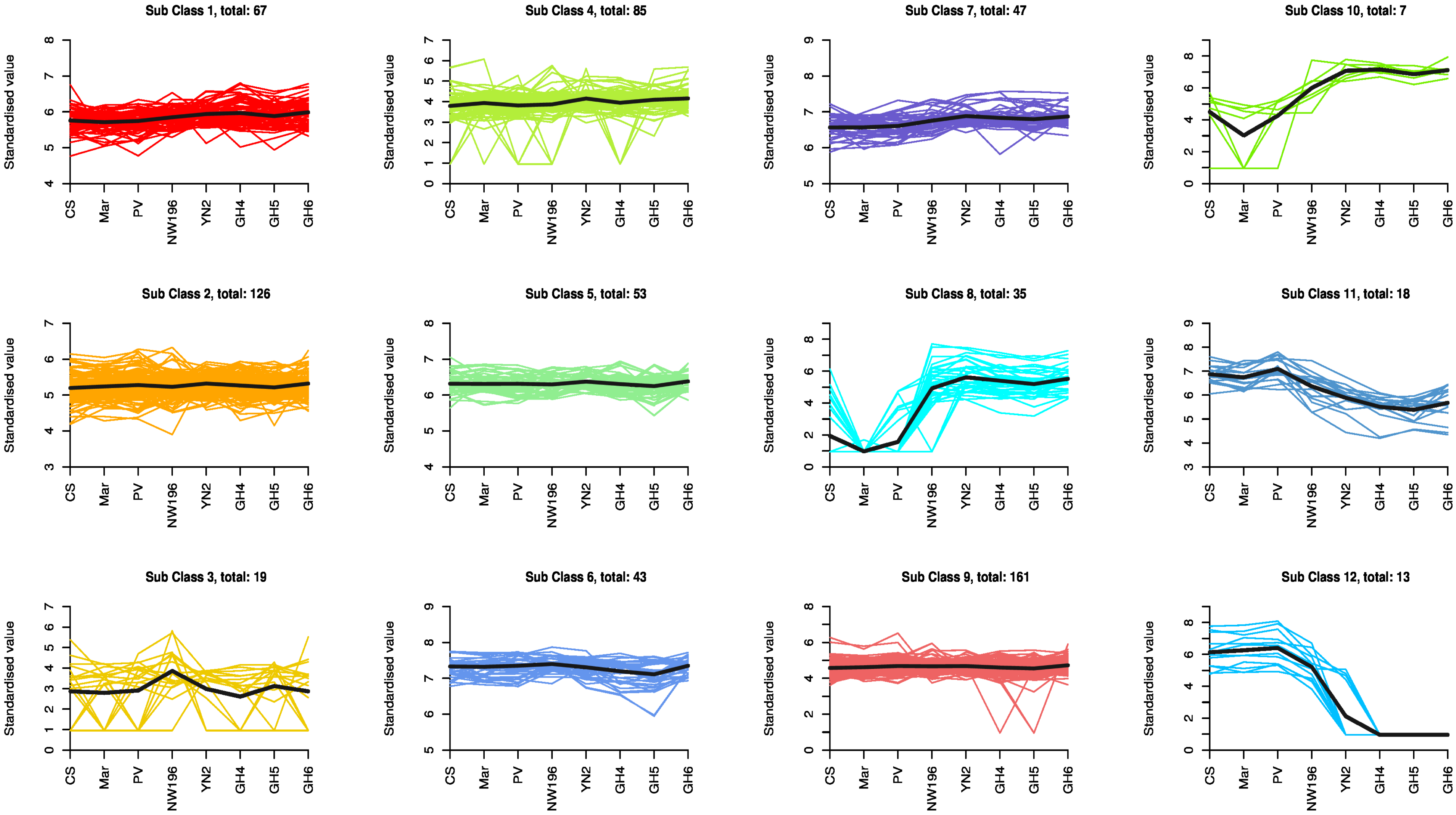

Supplementary Figure 5. K-means clustering analysis of patterns of 674 metabolites in eight grapes
